# The Fatal Camel

**Published:** 1893-04

**Authors:** 


### THE FATAL CAMEL.

The popular conception of the camel makes him out to be an ugly, an unamiable, but on the whole a useful creature. A paper which Mr. E. A. Floyer, Inspector-General of Egyptian Telegraphs, has published in the *Kew Bulletin*, will go far to remove this impression. The country between the Nile and the Red Sea is well known to be nowadays a dreary desert; it is equally well known that less than 2,000 years ago it was able to support large troops of roving cavalry, who picked up their living on spots that would now starve a lizard. In the same way Palestine, where we read of thousands of chariots and horsemen moving about in Biblical times, is now in great part a barren waste. As far as the Egyptian desert is concerned, Mr. Floyer believes that it is the camel and his Arab owner that are in fault. Originally the valleys must have abounded with trees ; indeed, their Arabic names still testify to this. As long as the Arabs were confined to these valleys, they took care of the trees for feeding the camels. But by degrees they got a footing in the Nile Valley; they hired their camels out to farmers, and when they returned for brief visits to their home valleys, they let the camels gorge their fill on the leaves and young shoots that in former years were carefully protected. Your camel is a greedy brute, and says Mr. Floyer, your Arab is not much better. Not content with letting his camel eat the edible portion of the trees, he proceeded to cut down the remainder and convert it into charcoal for sale to the farmers. Thus the land was gradually cleared, and in natural sequence it became the waterless desert it now is. The camel can hardly be called the Ship of the Desert so rightly as the creator of it.

